# Clinical metagenomics: ethical issues

**DOI:** 10.1099/jmm.0.001967

**Published:** 2025-02-27

**Authors:** Tess Johnson, Euzebiusz Jamrozik, Prashanth Ramachandran, Stephanie Johnson

**Affiliations:** 1 Ethox Centre, Oxford Population Health, University of Oxford, Oxford, UK; 2 Pandemic Sciences Institute, Nuffield Department of Medicine, University of Oxford, Oxford, UK; 3 Department of Infectious Diseases and Royal Melbourne Hospital Department of Medicine, University of Melbourne, Melbourne, Australia; 4 Monash Bioethics Centre, Monash University, Melbourne, Australia; 5 Department of Infectious Diseases, Peter Doherty Institute for Infection and Immunity, University of Melbourne, Melbourne, Australia

**Keywords:** clinical ethics, diagnostic, ELSI, metagenomics, next-generation sequencing

## Abstract

Metagenomics is increasingly used for diagnosis in hospital settings. It is useful particularly in cases of unknown aetiology, where novel or difficult-to-diagnose pathogens are suspected, and/or following unexplained disease outbreaks. In this paper, we present three use cases that draw on existing reports: one involving a patient in intensive care with encephalitis of unknown aetiology; a second case with likely infection with drug-resistant *Klebsiella pneumoniae* and an incidental finding of unknown relevance; and a third case situated in an unexplained outbreak of acute hepatitis in children, with severe outcomes due to co-infection. We examine each case in turn, highlighting ethical questions arising in relation to clinical issues including: disclosure to patients of untreatable disease, cost-effectiveness, the value of resistance testing, sensitivity and specificity, uncertain or unexpected findings, patient consent and data sharing. We conclude by proposing recommendations for further research and developing particular pieces of guidance to improve clinical uses of metagenomics for diagnosis.

**Impact Statement**

This article adds an ethics lens in the area of clinical uses of metagenomics for patient diagnosis. There are no thorough ethical investigations that focus specifically on diagnostic use of metagenomics to date, making this article the first of its kind in the area. We use current paradigmatic use cases to detail our ethical analysis, which grounds the work in real-world applications of metagenomics. By proposing recommendations for future research and guidance development, we aim to support appropriate use of metagenomics, ultimately improving diagnostic accuracy and patient outcomes.

## Introduction

Metagenomics is a pathogen-agnostic sequencing method that is increasingly used for diagnosis in hospital settings, particularly in cases of unknown aetiology, where novel or difficult-to-diagnose pathogens are suspected, and/or following unexplained disease outbreaks. Clinical metagenomic testing may complement the existing microbiological diagnostic methods for some diseases [1], with proponents arguing that its routine implementation as a diagnostic method could cause ‘a dramatic paradigm shift in microbial diagnostic testing’ (p. 321) [2].

In Box 1, we present three potential use cases for metagenomics in hospital settings, which draw on existing literature to varying extents (Case A [3], Case B [4] and Case C [5]).

Box 1. **Three cases of diagnosis using metagenomics**

***Case A:**Patient A has been re-admitted to hospital with clinical evidence of encephalitis. He is now in ICU with more severe symptoms. Infection is suspected as the underlying cause, but standard microbiological tests have come back negative. His clinicians send cerebrospinal fluid for metagenomic analysis. Results demonstrate a central nervous system enterovirus infection. With no specific treatment for his viral encephalitis, the clinicians provide ongoing supportive care. His condition deteriorates, with evidence suggesting a poor neurological outcome. In a discussion with his family, a decision is made to switch to palliative care.*

***Case B:**Patient B has arrived at the emergency with a fever, respiratory symptoms and sepsis. A sputum sample is sent for culture and metagenomic analysis. Before sputum culture and sensitivities are completed, metagenomic results identify* Klebsiella pneumoniae *with a bacterial genotype associated with extended-spectrum β-lactamase (ESBL) production. Clinicians increase infection control precautions, cease treatments likely to be ineffective for ESBL infections and switch to carbapenem therapy. The metagenomics results are also positive for* Tropheryma whipplei, *which appears to be unrelated to the current clinical presentation.*

**Case C:**A hospital begins to see a rise in cases of acute hepatitis of unknown cause in children. Patient C, a child, is in ICU with hepatic failure and is waitlisted for a liver transplant. Tests for hepatitis A–E and multiple other causes of viral or autoimmune hepatitis are negative. The child’s parents consent for a blood sample to be taken for metagenomic analysis. The analysis reveals the presence of human adenovirus 41F. Adeno-associated virus 2 is also identified, indicating a potential role for co-infection in severe outcomes. Metagenomics of similar cases confirm the presence of these viruses in multiple others and also find an association between human genotype HLA-DRB1*04:01 and severe disease.

Many of the important questions raised by the implementation of new technologies such as metagenomics for diagnosis have technical, operational, and also ethical dimensions. Important questions that involve ethical considerations might include, for example: under what circumstances should metagenomics be preferred to diagnosis *via* traditional methods of culture, antigen or antibody testing, or PCR? Which patients should receive metagenomic testing, and is it worth paying for? What types of findings should microbiologists report to clinicians, and when should they withhold information? Which results should clinicians report to patients? What are potential difficulties with patient consent?

In this paper, we aim to identify and explore the most pressing ethical issues arising in the context of pathogen metagenomics, as well as to provide initial recommendations for further research or guidance development to ensure ethically acceptable implementation of metagenomics in routine clinical use. Our analysis is limited to hospital settings. We leave public health metagenomic surveillance out of this discussion, as these uses may raise distinctive ethical issues. We also limit our analysis to questions of clinical ethics concerning metagenomics as a diagnostic tool—that is, although we note where individual diagnostic information could be useful for outbreak investigation purposes, these uses for public health purposes may warrant a separate analysis [1,6].

Our analysis aims to be practically orientated and useful to real-world decision-making. Therefore, we use Cases A–C (Box 1) to ground and guide our analysis. We analyse each case in turn, exploring relevant ethical issues. Where relevant, we consider and take lessons from the ethical issues raised in the related area of human genomics, since human genomics has been a major topic of ethical analysis in recent decades, and some issues are common across human and pathogen (meta-)genomics.

## Ethical issues highlighted by Case A: viral encephalitis

Case A is already a reality in some hospital settings, where metagenomics is becoming a frontline diagnostic tool in the investigation of encephalitis of unknown aetiology [3]. Standard microbiological workup of cerebrospinal fluid (CSF) samples may include culture, antigen testing or PCR. There are several cases in which metagenomics may complement or replace existing diagnostics workflows. First, in rare cases where other tests are inconclusive, metagenomics may allow the diagnosis of microbes that are difficult to culture, have not been considered by clinicians, or for which PCR is not available or produces false negative results [7]. Second, metagenomics may offer value in replacing the multiple other testing methods required to detect viral, bacterial and eukaryotic pathogens with its single, pathogen-agnostic test. Third, metagenomics may pose a particular advantage compared to traditional methods like culturing or PCR when it comes to viruses, due to the difficulty of traditional culture methods and the potential to identify organisms for which PCR testing is not (readily) available [8]. Finally, metagenomics is beneficial for its negative predictive value, especially in regions where autoimmune encephalitis is in roughly equal circulation alongside viral encephalitis: with opposing treatment regimens, ruling out viral infection quickly *via* metagenomic testing can allow for earlier, more appropriate treatment of non-infectious causes.

### Disclosure of pathogens identified by metagenomics as causal agents

Clinicians may be unprepared (and may fail to prepare their patients) for the kinds of results they may receive from clinical metagenomics [8]. Metagenomics shows the presence of microbial DNA or RNA, and clinical judgement is necessary to determine the extent to which positive testing reflects any likely infectious cause(s) of disease. For Case A, CSF testing has the advantage of being a sterile site, where no microbes would be expected in healthy patients, whereas at non-sterile sites, the clinical relevance of detected pathogens can be even more difficult to establish.

Only some of the microbes detected by metagenomics (especially at non-sterile sites) will be clinically relevant, and experience with other microbiological testing suggests that the detection of microbes sometimes leads to over-diagnosis and over-treatment of microbes not currently causing disease. The over-attribution of causal power to microbes, or ‘microbial determinism’ [9], has an analogue in human genomics: genetic determinism. Genetic determinism is based on the sequence hypothesis: that genes, transcribed into RNA, encode proteins, which shape biological development, even up to as complex traits as behaviour. In reality, interactions between genes and environmental factors make the process far more complex. Similarly, microbial determinism is sometimes based on simplistic versions of germ theory, for example the idea that “host plus germ equals disease” [10]. Addressing microbial determinism may be especially important to reduce reliance on ‘magic bullet’ treatments and ‘technological fixes’ for infectious diseases such as antibiotics by both clinicians and patients alike. Clinicians will need to consider how to apply such norms to metagenomics, given the large amounts of genomic information about microbes produced by such approaches.

A classification (pathogenic, possibly pathogenic and benign) of disclosable microbial findings should be further developed for the metagenomics context and might guide clinicians in determining what information to disclose to patients in cases like A. For instance, a finding of human immunodeficiency virus (HIV) might be classified as a clearly disclosable finding (after or alongside confirmatory testing, if required); a finding of cytomegalovirus (CMV) might be classified as only possibly pathogenic and not necessarily requiring disclosure. Such a system should sit alongside existing predefined reporting thresholds that aim to avoid attribution of pathogenicity to organisms present in a sample as part of background commensal flora or environmental contamination. The classification list would then apply for pathogens detected at levels above the threshold expected for background contamination. However, clinicians will still need to use their judgement as the clinical relevance of certain organisms (even those considered benign) may vary based on the clinical context.

### Untreatable diseases/medical futility

Many potential causes of encephalitis lack specific treatment, including enteroviruses [11], which may limit the prospect of direct therapeutic benefit to a patient from a confirmed diagnosis. However, patients and their families may benefit from diagnosis using metagenomics given the peace of mind they gain from a firm diagnosis and/or improved prognostication in some cases [12]. Although CSF standard testing including PCR will often detect relevant enteroviruses, metagenomic testing may provide a diagnosis when PCR produces a false negative, when unusual infections are involved, in cases of co-infections, or novel CSF infections. There may also be benefits in the cessation of (potentially harmful) treatments for other differential diagnoses, such as antivirals (e.g. for herpes simplex encephalitis) or steroids (e.g. for autoimmune causes). Particularly in cases where metagenomics informs differential diagnosis where the cause of encephalitis may be either autoimmune or infective, the value of metagenomics in helping clinicians avoid starting inappropriate treatment may be significant, for example where steroids would worsen the morbidity related to viral encephalisti.

There may be public health benefits of testing for some transmissible but untreatable diseases where further infection control measures can prevent transmission (although this is unlikely to be feasible for enteroviruses such as in Case A). In any case, the lack of direct clinical benefit may raise ethical questions regarding cost-effectiveness, medical futility, and appropriate rationing of expensive diagnostic methods including metagenomics. Further, clinicians may expect not to receive the results in time to take appropriate action [2]. Although available techniques can offer 24-h turnaround times, currently average turnaround times often sit around 48 h and depend on laboratory capacity [13]. Narrow views of medical futility consider only therapeutic benefit, of which there seems to be very little or none for Patient A. However, we might also consider other benefits that may improve quality of life, such as finding peace of mind following diagnosis and prognosis. These reasons might speak in favour of the use of metagenomics for patients with undiagnosed encephalitis.

There is an interesting parallel here between Case A and issues of untreatable disease findings in human genomics. An untreatable disease is sometimes uncovered through human genome sequencing, leading to questions about futility, rationing and whether testing should be performed and reported to begin with. Where there is a known hereditary disease like Huntington’s disease within a family, the patient and/or family may wish for testing to provide confirmation, and a patient’s positive result will be anticipated. As a result, the patient and family may be prepared for the diagnosis [12]. In contrast, some patients may prefer not to know their diagnosis and may, therefore, decline genetic testing for themselves or genetic testing of a gamete, embryo or foetus to ensure an unaffected child [14]. Similarly, while some families may strongly support metagenomic testing for presumed infectious encephalitis, others might decline for various reasons. Where there is minimal therapeutic benefit and no likely public health benefit, declining metagenomic testing may be an important patient prerogative. This raises questions surrounding whether patients will have the opportunity to be informed of testing and to decline the option of testing in advance, which is not standard for many existing clinical microbiological diagnostic tests.

### Rationing and cost-effectiveness

Rationing and cost-effectiveness are issues also raised in this case. Rationing may be necessary in resource-limited settings, where the lack of clinical benefit might be one reason for allocating metagenomics for diagnosis to a different patient than Patient A who is also in need. Wider implementation of metagenomic testing may also require appropriate funding models for the laboratories that provide such testing, especially while assays remain expensive and/or significant microbiologist time is required for verification and communication of results.

Metagenomic testing may become increasingly cost-effective as assay costs come down over time or when certain aspects of metagenomics reporting can be standardized and automated. Given the greater cost of metagenomics compared to other molecular diagnostic methods such as PCR, it may not be cost-effective as a first line test in cases like Case A—particularly if there is already a good reason to suspect a particular aetiological agent for which targeted PCR can be performed. A relevant caveat is that where there are multiple or rare pathogens suspected, multiple tests or assays may be needed for diagnosis, meaning more cost and time overall for complex cases. However, metagenomics is currently costly both in terms of hardware and set-up, maintenance and consumables, specialized staff and the development of pipelines for analysis [5]. Whether its costs are justified depends on its marginal benefit over other, less expensive methods, and one point for further consideration is that given the volume of potential incidental findings, wider implementation may require recruitment and training of additional microbiology staff (as well as training for bedside clinicians). For ethical and effective routine implementation of metagenomics as a diagnostic method, more guidance is needed on its typical cost-effectiveness and appropriate implementation, especially in more resource-limited settings.

## Ethical issues highlighted by Case B: respiratory metagenomics

While metagenomic testing is not widely available for the diagnosis of acute respiratory infections, pilot studies have examined the potential for fast turnaround of metagenomics results in such contexts [4]. In a study of patients with suspected lower respiratory tract infection, metagenomic testing resulted in same-day diagnosis for 86% of samples (*n*=110), and testing led to changes to antimicrobial treatment in nearly half of all same-day diagnoses (48%, *n*=53). The authors conclude that respiratory metagenomics ‘has the potential to become a ﬁrst-line test for severe pneumonia’ [4] (p. 165).

In our slightly different case, Patient B is found to have a bacterial cause of respiratory infection. *Klebsiella pneumoniae* may be considered the most likely cause of both respiratory symptoms and sepsis in Patient B based on metagenomic results. Metagenomics also reveals the presence of a bacterial gene for extended-spectrum *β*-lactamase (ESBL), so clinicians may decide to avoid first-line antibiotics for pneumonia in favour of other antibiotic treatment options such as carbapenems. This shows an additional potential therapeutic benefit of metagenomics, in that it can help in the tailoring of treatment options for a patient where drug resistance is predicted.

However, the case is complicated by additional findings of uncertain clinical relevance. While Patient B has not reported any symptoms aligning with a diagnosis of Whipple’s disease, metagenomics also detects *Tropheryma whipplei* . Whether the patient should be notified of (and perhaps treated for) this microbe is uncertain, since no trials have addressed the treatment of incidentally diagnosed carriage of this organism.

### Diagnostic benefits and resistance testing

Case B highlights ethical questions regarding tailoring the treatment of drug-resistant infections, the sensitivity and specificity of testing, and the problems of uncertain findings and/or false positives. First, there are clear benefits to patients from the ability to tailor treatment according to metagenomic results. In this case, clinicians using the data can make more informed decisions about the treatment of ESBL-producing *K. pneumoniae,* such that they avoid ineffective treatment while the patient’s condition only becomes worse.

Genomic drug resistance testing using molecular assays is recommended by the World Health Organization (WHO) for HIV and tuberculosis (TB) in areas with high prevalence [15]. However, in contrast to sequencing of resistance genes within the genome of a pathogen isolated in a laboratory culture, in metagenomics, the association between a particular pathogen among the microbes present in the sample and a resistance gene found may be less certain (e.g., because a resistance gene may be present in a sample but this gene may not be present or active in the pathogen causing the current episode of disease). This leaves open the possibility of a pathogen being treated as resistant and clinicians seeking second-line treatment, when in fact, the resistance detected was associated with another microbe (e.g. in commensal flora), and first-line treatment would have been effective.

More generally, the detection of bacterial genes for ESBL may not always correlate with phenotypic resistance in the organism that is presumed to be the cause of the current disease episode. First, as above, the ESBL production gene may be carried instead by other bacteria in the sample (through environmental contamination or through presence in the microbiome) or it may be in a mobile genetic element. In patients whose illness is caused by different bacteria that may not carry these genes, clinicians will want to avoid opting for second-line antibiotics over the first-line antibiotics to which the target infection may still be sensitive. Second, while the detection of ESBL-carrying bacteria is strongly correlated with resistance rates to common beta-lactam antimicrobials [16], genotype and phenotype do not always correlate. Thus, while it may be appropriate to use carbapenems in unwell patients where ESBL has been detected (*via* metagenomics or other methods), such therapies can be appropriately de-escalated if later phenotypic testing (i.e. culture and sensitivities) shows less bacterial resistance than initially expected or identifies an alternative causative organism. Further, it may also be appropriate to de-escalate infection control measures given additional results in some situations (e.g., those that suggest that the patient is unlikely to spread the resistant bacteria they carry). Given the evidence of negative effects on carriers of resistant infections from some infection control measures, such de-escalation may be ethically required [17].

There may also be public health benefits to population-scale improvements in the tailoring of treatments, including reductions in the prevalence of antimicrobial resistance. Metagenomics has a distinct advantage here over other microbiological methods such as culturing, for which separate testing must be done to determine drug susceptibility, increasing turnaround time on test results that could inform treatment. There are also potential benefits to clinicians and other patients in the hospital from this testing, insofar as infection control methods implemented in response to such findings might protect clinicians and other patients from infection.

### Sensitivity and specificity

Specialised guidelines have been developed for metagenomic reporting as part of research[18; 19]. Existing guidelines such as the STARD reporting [20] aim to ensure that the introduction of new medical diagnostics is based on accurate and thorough reporting on factors such as their sensitivity and specificity as compared to the existing clinical reference standard. These guidelines may need to be adapted for the inclusion of metagenomic diagnostics in clinical microbiology reporting in clinical (and research) settings. The pilot study on which Case B is based showed additional benefits of metagenomic testing in terms of increased sensitivity and specificity compared to other methods [4]. This rapid metagenomic testing of patients with lower respiratory tract infection was able to produce a same-day diagnosis in many cases, including diagnoses that led to changes in antimicrobial treatment [4]. Multiple other studies support metagenomics’ greater sensitivity and specificity under certain conditions [2,8]. With high sensitivity, metagenomic analysis is more likely to identify an organism, given its presence in the sample. Because metagenomics is pathogen-agnostic, it may in some cases outperform more targeted methods. However, its sensitivity is somewhat limited by the sequencing depth (number of times a fragment is sequenced in a sample) required to detect the presence of microbial DNA, given that over 99% of a sample is often human DNA [2,20]. Deeper metagenomic sequencing with appropriate management of human genomic data may, nevertheless, improve diagnosis of common pathogens for which reference databases are highly accurate [2]. Greater sensitivity of deeper sequencing raises a trade-off, though: the risk of picking up organisms that are environmental contaminants or normal flora, with the potential for incorrect attribution of a causal role in a clinical infection, leading to false positives. Further, sequencing at increased depth is expensive and may reduce the cost-effectiveness of metagenomics, presenting a trade-off between sensitivity and cost that needs further exploration. In Case B, sputum sequencing is sensitive enough to confirm the presence of *K. pneumoniae,* leading to the clinical benefits discussed when this is attributed as the causative agent, but in future more widespread use metagenomics may end up complementing rather than replacing other testing modalities.

The use of metagenomic testing in Case B also raises more specific ethical issues concerning the interpretation of the raw data collected and false positive results. As other authors have suggested, interpreting metagenomic results is complicated by the complexity of the human microbiome and how this interacts with the sensitivity of the approach. As a result, it can be ‘difficult to distinguish between pathogens, bystanders, commensal flora, and contaminants’ [5] (p. 15). There are several different issues with the interpretation of a positive result of metagenomic testing. First, if the cause of the disease is misattributed to a non-pathogenic microbe in the sample (perhaps bacteria that are part of the commensal flora), then this would be considered one type of false positive. The consequences of such false positive misattributions in a case like B can include inappropriate or ineffective treatment against the wrong microbe instead of *K. pneumoniae*, prolonged hospital stays, or increased risk of morbidity or mortality for the patient.

A second type of false positive relates to the detection of a microbe that is not actually in the sample. This has been documented [4] and can lead to unnecessary further clinical investigation, inappropriate treatment where the symptoms match the non-present microbe or other consequences. For instance, there may be cases where fragments are similar enough to diseases that are reportable to public health authorities. This was reported when metagenomic analysis indicated the presence of a notifiable zoonotic pathogen as the suspected cause of disease [4]. Government organizations became involved when the individual sought treatment at a clinic, and by checking the sequences, it was found that a mistake in the reference database had resulted in the false positive for a sequence that did not in fact match the notifiable zoonotic pathogen. This indicates a potential problem with the specificity of metagenomic testing, with microbes that are not in the sample being identified. The ethical implications of false positives for notifiable diseases may be significant where public health resources are scarce or where such results are reported in the media. The individual may also be harmed, again, by receiving inappropriate or ineffective treatment (or being subject to isolation orders).

### Uncertainty and additional or incidental findings

Metagenomic testing may identify organisms that are likely not relevant to the current disease state. In defining incidental findings, we might separate out two kinds of cases here: (i) cases where clinicians were not searching for the organism that was discovered, and (ii) cases where the clinician receives a positive result for the type of organism they were searching for, but they are uncertain as to the significance of the positive result. For example, a clinician may find a potential pathogen like *Clostridioides difficile* upon bowel testing. This may not be an incidental finding, if the clinician was searching for a potential pathogen in that organ system, and so a finding of *C. difficile* may have been expected, not incidental, in one sense. However, it may be an incidental finding in the sense that the clinician cannot be certain that the *C. difficile* is causing the current episode of disease in the patient, or was expecting it to be potentially present but not likely to be causing the current clinical syndrome.

Some incidental findings may be potential pathogens—in most cases, they may be carried asymptomatically by healthy individuals but in rare cases might cause disease (either in the short or long term). As shown in Case B, the detection of organisms such as *T. whipplei* may be particularly challenging, given the potential for asymptomatic carriage [21], the long lag time between infection and symptoms among those who do develop disease [22] and the lack of a single readily recognizable clinical syndrome [23]. Similarly, low abundance viral pathogens can often be detected in inflammatory samples regardless of whether the patient had an infectious aetiology or not. The significance of these at low levels is not clear, yet whether these reads align with the clinical syndrome is important for interpretation [24]. Where there is uncertainty about the veracity or clinical relevance of a finding, microbiologists and clinicians may face difficult ethical decisions. Should such results be reported by microbiology labs to clinicians or by clinicians to their patients? Which detected microbes should be treated? What course of action will pose the least potential harm to the patient? Appropriate adaptation of existing guidlines such as STARD, particularly regarding the reporting of polymicrobial findings, will require both scientific and ethical analysis and may draw upon similar approaches in human genomic reporting.

If an incidental finding is represented as a potential cause of the pathology, there is a possibility that clinicians will undertake inappropriate treatment, as discussed in greater detail in the analysis of Case A. Another concern may be the role that clinicians’ assumptions and biases may play in assessing possible aetiological agents from metagenomic analysis and attributing the cause of disease, particularly under conditions of uncertainty. Where there is a wide range of possible causes identified, or where rare pathogens are detected, there may be more room for the influence of clinician biases. This might include situations where metagenomics identifies a pathogen that may be causally linked to the patient’s clinical syndrome, but where this syndrome is not recognized by doctors as being associated with the pathogen in question. In such situations, clinicians might incorrectly assume that the result is a false positive, meaning that patients may sometimes miss out on potentially beneficial treatment.

These issues of additional and incidental microbial findings have received some attention in the literature [8,12]. Hall *et al*. propose that unwanted findings can be avoided with a technical fix, by filtering out a panel of high-consequence pathogens [8]. However, relevant findings can sometimes be entirely unexpected, and their clinical relevance can be uncertain. Magiorkinis *et al*. instead favour a strategy of consultation with the patient and the family, who may also be considered relevant stakeholders, for consultation on reportable pathogens that might include, in their view, HIV, hepatitis B and C and human T-lymphotropic virus [12]. In Case B, *T. whipplei* is highly unlikely to be the aetiological agent, considering the patient’s symptoms and the identification of *K. pneumoniae* through metagenomic testing. However, the knowledge may cause Patient B anxiety about their risk of Whipple’s disease, which (although rare) can be fatal [22]. As such, the clinician faces the question of whether to inform the patient about the result or treat it with the goal of eradicating this potential pathogen. As we discuss in relation to Case C below, similar issues may arise regarding sharing of metagenomic results by microbiologists with clinicians.

Again, there may be relevant parallels here with human genomics. Study participants in genomic research have reported feeling disillusioned with the technology (as its tood in 2014) based on the frequent inaccuracy of findings [25]. Ethical frameworks for dealing with uncertainty and possible inaccuracy of results as proposed in the human genomics setting [26] may also be useful for decision-making surrounding uncertain results of metagenomic testing.

However, one important disanalogy here between human genomics and metagenomics is the timeframe and context of clinical urgency in which the human genome as opposed to pathogen genomic results may be interpreted. While patients in a human genetic testing clinic may have time to discuss the plan for and results of genomic testing with a genetic counsellor, as well as with their family and others, patients admitted with sepsis do not have the same opportunities or luxury of time. It may, therefore, be appropriate for clinicians to inform patients about testing and make time-sensitive decisions on the most immediately relevant results and for less time-sensitive results to be considered and discussed later. More training for clinicians and resources for infectious disease units may be needed given the specialist knowledge and communication skills that may be required for appropriate patient disclosure.

## Ethical issues highlighted by Case C: severe acute hepatitis outbreak in children

Case C is based on an outbreak of severe acute hepatitis of unknown aetiology in children in early 2022 [5]. In April 2022, WHO announced the provision of additional support alongside the European Centre for Disease Prevention and Control for ongoing investigations [27]. By July 2022, 35 countries in 5 WHO regions had reported cases, and WHO launched a global online survey to estimate comparative incidence and to consider possible causes [28]. Metagenomic evidence proved key in supporting the hypothesis that co-infection had led to severe outcomes. There was a high prevalence of human adenovirus 41F across the cases, which was unexpected, given that human adenovirus 41F is usually associated with self-limiting gastroenteritis in children.

There was also metagenomic evidence of adeno-associated virus 2 as a driver of more severe illness when present as a co-infection with human adenovirus 41F [29]. Researchers have suggested that it was co-infection with these two viruses that led to the symptoms of immune-mediated liver injury seen in the children who consequently needed liver transplants, not all of whom survived [5]. Such work is important because, without a diagnosis, patients with unknown disease aetiology face a higher risk of delayed or inadequate treatment, re-admission, and increased morbidity and mortality [2].

Finally, metagenomic testing also revealed a human host genotype associated with severe disease – in this case, HLA type was associated in some children with increased vulnerability to severe liver injury [5]. Human DNA is usually filtered out in the bioinformatics stage of metagenomic testing. However, clinicians and researchers may be interested in human data insofar as this may aid diagnosis. Host DNA data can also be used in new ways to look for genetic signatures of cancerous human cells where these might cause symptoms overlapping with infectious causes [30]. Case C demonstrates that human DNA reads may be clinically relevant even where the main goal of metagenomics is the identification of potential pathogens. Laboratories and clinicians, therefore, need to develop policies regarding when and how human genomic data might be appropriately used in the context of clinical metagenomics.

It may have been more difficult to identify the causes of hepatitis in these cases without metagenomics. Although PCR may detect adenoviruses like 41F, it is unlikely that all the relevant contributors to the disease, including multiple infections and a human genetic risk factor, would have been identified as efficiently by standard testing approaches.

### Data sharing for public health uses of metagenomic data

Case C raises several ethical questions about consent to data collection and the scope of data sharing. There is a strong ethical case for local and international data sharing during epidemics. However, there is also an ethical tension between protecting the privacy of infected individuals and sharing data to improve understanding of and response to an outbreak. Deciding which data (and/or metadata) to share and what level of detail is appropriate requires both scientific and ethical judgements. Such decisions may also be legally complex where different regulations apply (e.g., where both pathogen data and human genome data outputs of metagenomics are shared during international epidemics). There remains a need for additional work to enable ethically appropriate data sharing during epidemics, including capacity building for microbiology laboratories and public health agencies.

Beyond the sharing of data for public health purposes, we must also consider patient consent to data collection, particularly in the case of children. In many countries, clinicians are not required to obtain consent for separate specific diagnostic tests such as metagenomic analysis or for secondary analysis for public health purposes. However, in the real-world counterpart of Case C, data collection was performed for research as well as public health reporting into the aetiology of severe acute hepatitis in this 2022 outbreak. UK clinicians and researchers therefore obtained parental consent for data collection for research.

In the absence of research being performed, clinicians would not have been required to obtain parental consent. This is because according to Reg. 3 of The Health Service (Control of Patient Information) Regulations 2002 [31], clinicians in the UK can collect and process patient confidential information for national surveillance of communicable diseases, and similar rules apply in many other countries. According to WHO guidance on the ethics of public health surveillance, individuals have an obligation to contribute to surveillance to promote the public good [32].

However, widely endorsed, detailed norms of appropriate uses of surveillance data are yet to be developed, which may be unsurprising given the rapid increase in the availability and accuracy of pathogen genomic testing (including metagenomics). For example, public health researchers often receive a waiver of consent (or even a waiver of ethics review) to study and publish analyses of outbreaks, including in academic journals. It is yet to be determined whether this results in a decline in ethical standards for such research (compared to prospective research ethics reviews of similar studies where public health waivers do not apply). There may also be special cases proposed in which a collective interest in data processing that can be expected to result in substantial societal benefit implies the existence of a duty for individuals to share their data (for an equivalent in the human genomics context, see [33]). Researchers should in any case take special care upon publication, for example, to ensure that patients are not (re-)identifiable, especially where they have not given consent for the analysis of their clinical samples.

### Informed consent

In cases where individual patient consent is deemed necessary (perhaps because there is no public health justification for surveillance testing or because the findings will primarily contribute to research), we might still ask about the degree to which patient consent given to metagenomic diagnosis can be considered adequately informed.[12] Alongside broad issues concerning understanding the kinds of information produced *via* metagenomics, it is unlikely that a patient (or their parent) would anticipate the uncovering of incidental or additional findings in the infectious disease context [12]. Given the wide range of possible disease findings from metagenomics, can patients truly be adequately informed of all possible eventualities in the case of various microbes that might potentially be identified *via* metagenomics? In this context, risks and benefits should be discussed with patients concerning the reporting of various types of findings [12].

The appropriate approach to consent for metagenomic testing may also draw on frameworks discussed in human genomics. For example, whole-genome sequencing is sometimes offered for paediatric cancer diagnosis, and there are guidelines produced to help parents understand the implications of whole-genome sequencing for their child [34], including the connection between genetic predisposition and disease, and the relational nature of genomic data that may reveal predispositions of family members as well [35]. Some of these issues may cross over to metagenomics, as well. For instance, while there are no direct implications of metagenomic findings for genetic relatives, other kinds of relational data (such as infections that may be spread between people) are produced and can create risks surrounding privacy and confidentiality. Systems must be developed for discussing such risks and implications of metagenomic testing with parents.

### Sharing metagenomic laboratory results with clinicians and patients

Beyond individual consent to data collection, ethical questions surrounding the use of metagenomics in Case C also arise regarding data-sharing between labs, clinicians and patients. Metagenomic analysis usually occurs within a specialist microbiology team, who must decide how much of the information to share with clinicians and in what format. Clinicians, in turn, must decide how much information to share with patients (or their parents).

We have already discussed issues with findings of untreatable disease (Case A) and findings of uncertain accuracy or clinical relevance (Case B). But, more generally, how should microbiologists decide how many and what kinds of metagenomic findings to report to clinicians? (And how might limited information sharing between microbiologist and clinician affect the sharing of relevant health information by the clinician with family members or close contacts of the patient?) Microbiologists already use significant judgement and experience in determining what to report to clinicians, for example, regarding antimicrobial susceptibility testing (e.g., because censoring certain antibiotic sensitivity results can prevent inappropriate use of antibiotics by clinicians). Such judgements may become more complex because of the nature of the data gathered through metagenomics. Microbiologists will at least in part determine whether to report the presence based on the reliability of the methods, the rarity of the microbe and, thus, the reliability of reference database data and likelihood of environmental contamination of samples before sequencing [8]. While microbiologists’ interpretation and discretion with reporting should ideally occur within the framework of objective thresholds determined when receiving laboratory accreditation, current threshold-setting guidelines based primarily on the abundance of reads in a sample may miss low-abundance causative agents, e.g., in cases of pulmonary tuberculosis [36]. For this reason, such thresholds may also require review.

Indeed, researchers in metagenomics have found that ‘when collaborators are presented with the taxonomic report from a metagenomic study for the first time, they may be overwhelmed’ [8] (p. 4). One might expect a similar response from clinicians if presented with data from more standard microbiological testing without prior processing by laboratories. For example, labs routinely do not report the growth of bacteria that are not likely to be clinically relevant (e.g., reporting ‘mixed growth’ in urine or sputum samples without specific microbes or sensitivities listed), which, from a microbiologist’s perspective, may reduce the risk of clinicians making treatment decisions based on irrelevant information. There may be an equivalent acceptable means of censoring or summarizing metagenomics results that allow microbiologists to employ their judgement in determining how to report results back to clinicians.

In Case C, metagenomic analysis led to the hypothesis that co-infection with adeno-associated virus 2 played a significant role in increasing the severity of symptoms resulting from infection with human adenovirus 41F. However, determining that such results are significant requires careful analysis—not all detected microbes will be causes of disease. Microbiologists may fear that by sharing a wide range of microbial data, they may increase the chance that clinicians will inform patients and change treatments based on results that are unlikely to reflect the cause of the current disease episode, falling prone to the ‘microbial determinism’ discussed earlier. Metagenomic results may risk reinforcing common perceptions of the potential for microbes (or microbial genes) to influence disease states (or resistance to treatement) even where no such causal relationships are present.

## Conclusion

In this article, we have examined several ethical issues that arise when considering the implementation of metagenomics as a routine tool for diagnosis in hospital settings. These are outlined in Table 1, which presents the clinical metagenomic context, ethical question arising and recommendations for further ethics or scientific research, regulation, or tool development that is required to address the question.

**Table 1.** Clinical metagenomics for diagnosis, ethical questions arising, and recommendations for research and policy agenda along a diagnostic decision-making timeline

Clinical metagenomics context	Ethical question	Recommendations for research and policy
Clinician faces decisions about whether to offer metagenomic testing to a patient for a (likely) untreatable disease	Where attempting to treat a disease caused by a particular likely pathogen may constitute an inappropriate allocation of resources, should clinicians be allowed to offer metagenomic testing?	Develop frameworks to inform resource allocation at a higher level than clinician decision-making, drawing on existing frameworks for other expensive diagnostic testing methods
	Where attempting to treat a disease caused by a particular pathogen may be medically futile, should clinicians offer metagenomic testing?	Develop frameworks and training to inform clinician decision-making based on ethical considerations including medical futility alongside counterweighing considerations such as non-therapeutic benefits to patient and/or family and/or public health response
Clinician faces decisions about whether to offer metagenomics for the identification of resistant pathogens and tailoring of treatment.	Considering the likelihood of clinical benefit (which will depend on treatment options) and population benefit (which will depend upon the frequency of testing, the likelihood of the same resistance mechanism presence in the hospital environment, possibilities for containment etc.), should the clinician order metagenomics for resistance testing?	Resistance testing can have individual clinical and population-health benefits if it shapes treatment decisions; clinicians must be made aware of the increased risk of identifying carriage of resistant bacteria *via* metagenomic testing that may be unrelated to the current disease episode; beyond being guided by policy, clinicians must be aware of the risks to patient health of incorrect treatment tailoring based on misinterpretation of metagenomics results
Clinician faces decisions about when to disclose pathogens identified by metagenomics as causal agents of disease to a patient	Should clinicians share metagenomics findings where a likely disease-causing agent is identified with patients?	Development of a classification (pathogenic, possibly pathogenic, benign) and disclosable list microbial findings; training for clinicians to inform clinical judgement on disclosure based on specific patient context and likely causation
Clinician is faced with a decision about whether informed consent is needed for performing metagenomic testing on a patient’s sample	Should the clinician seek informed consent, and can consent ever be truly informed for metagenomic testing and the possible implications of disclosed results?	Risks and benefits of testing should be discussed with patients in advance, and where practicable, informed consent should be sought for testing where the purpose is diagnosis for patient benefit; particularly for those unable to consent (such as children) and the use of proxy consent: good frameworks exist in the human genomics space which may serve as appropriate examples for adapting to the metagenomics context; clinicians may require additional public health frameworks for considering the implications of results for others, given disease transmission information that may arise from testing
Clinician faces decisions on how to interpret a positive result from metagenomic testing	Given the potentially greater sensitivity and specificity of metagenomic testing compared to other methods, should the clinician rely upon the test results (and disclose them to the patient)?	Train clinicians to interpret results from metagenomic testing, particularly those regarding commensal, likely non-pathogenic flora, and those that may be the result of environmental contamination; develop (i) censoring systems in microbiology laboratories performing metagenomic testing, (ii) appropriate channels for microbiologist-clinician communication concerning microbes of concern and the possibilities of false positives, and/or (iii) in cases where incidental findings should be fed back to patient, develop systems for advance consultation with the patient (and family) concerning whether they would like to be informed about additional findings, particularly those that may be of high consequence; these systems might be developed drawing upon examples from human genomics consultation
Clinician is faced with incidental findings in addition to the causative agent of disease through metagenomic testing; they must decide whether to disclose these to the patient	Under what circumstances should clinicians disclose incidental/additional findings to patients, especially where the clinical relevance of these findings is questionable?	
Clinician is faced with a decision about how to share notifiable findings from metagenomic testing with public health authorities and/or others	How should the clinician disclose metagenomics findings to parties other than the patient, for reasons outside of benefiting the patient?	Clinicians should follow existing rules and regulations surrounding notifiable infectious diseases and obligations concerning disease surveillance in the public health interest; however, there may be a lack of guidance where metagenomic findings are complicated by questions of identifiability and the inclusion of human DNA in initial results; human DNA should be filtered out prior to sharing with public health agencies and efforts should be made to preserve patient anonymity
Microbiologist faces a decision about whether/what range of metagenomics results to share with clinicians	How much and which information should microbiologists share with clinicians, given their greater understanding of microbe pathogenicities, commensal flora, risks of false positives and environmental contamination that accompany metagenomic testing?	Develop protocols for sharing metagenomic results with clinicians, including training microbiologists to identify clinically irrelevant information, as for other types of testing

It is essential that efforts are made to address the ethical questions raised above before the widespread clinical use of metagenomics for diagnosis. Without resolving these issues, metagenomics may produce results that clinicians and patients are not well equipped to interpret. This could result in inappropriate treatment, prolonged hospital stays and giving patients information that they might not have wished to receive.

Further work is needed in many areas, and one fruitful area for learning comes from human genomics. Separate questions also remain concerning public health ethics analysis of the routine use of metagenomics for infectious disease diagnosis. We leave these areas for future work.

## Abbreviations

CMV: cytomegalovirus
CSF: cerebrospinal fluid
ESBL: extended-spectrum *β*-lactamase
HIV: human immunodeficiency virus
TB: tuberculosis
WHO: World Health Organization
